# Three dimensional echocardiography in congenital heart defects

**DOI:** 10.4103/0974-2069.41050

**Published:** 2008

**Authors:** Girish S. Shirali

**Affiliations:** Department of Pediatrics, Medical University of South Carolina, USA

**Keywords:** Atrioventricular septal defect, congenital heart defects, three dimensional echocardiography

## Abstract

Three dimensional echocardiography (3DE) is a new, rapidly evolving modality for cardiac imaging. Important technological advances have heralded an era where practical 3DE scanning is becoming a mainstream modality. We review the modes of 3DE that can be used. The literature has been reviewed for articles that examine the applicability of 3DE to congenital heart defects to visualize anatomy in a spectrum of defects ranging from atrioventricular septal defects to mitral valve abnormalities and Ebstein's anomaly. The use of 3DE color flow to obtain echocardiographic angiograms is illustrated. The state of the science in quantitating right and left ventricular volumetrics is reviewed. Examples of novel applications including 3DE transesophageal echocardiography and image-guided interventions are provided. We also list the limitations of the technique, and discuss potential future developments in the field.

## INTRODUCTION

Complex intra-cardiac anatomy and spatial relationships are inherent to congenital heart defects (CHD). Beginning over thirty years ago and until recently, the clinician's ability to image the heart by echocardiography has been limited to two-dimensional techniques.[1] In the interim, there have been important advances in two dimensional echocardiography (2DE). Improving transducer technology, beam-forming and miniaturization have led to significant improvements in spatial and temporal resolution using 2DE. However, 2DE has fundamental limitations. The very nature of a 2DE slice, which has no thickness, necessitates the use of multiple orthogonal ‘sweeps’. The echocardiographer then mentally reconstructs the anatomy, and uses the structure of the report to express this mentally reconstructed vision. This means that the only three-dimensional image of the heart is the ‘virtual image’ that exists in the echocardiographer's mind, and is then translated into words. It is not easy for an untrained - albeit interested - observer to understand the images obtained in the course of a sweep: expert interpretation is required. Since myocardial motion occurs in three dimensions, 2DE techniques inherently do not lend themselves to accurate quantitation.

Recognition of these limitations of 2DE led to burgeoning research and clinical interest in the modality of 3DE. Early reconstructive approaches were based on 2DE image acquisitions that were subsequently stacked and aligned based on phases of the cardiac cycle, in order to recreate a 3DE dataset.[2–5] While these approaches proved to be accurate, the need for time and offline processing equipment imposed fundamental limitations on their clinical applicability. In 1990, von Ramm and Smith published their early results with a matrix array transducer that provided real-time images of the heart in three dimensions.[6] While this was an important breakthrough, this transducer was unable to be steered in the third (elevation) dimension. Over the past five years, dramatic technological advances have facilitated the ability to perform live 3DE scanning, including the ability to steer the beam in three dimensions and to render the image in real time.[7]

## 3DE TECHNOLOGY

Technologic advances that have facilitated the maturation of 3DE techniques include the following:

- Matrix transducers
- Beam forming and steering in three spatial dimensions
- Display of three-dimensional information
- Software for quantification

### Matrix transducers

Two important advances in transducer technology, namely the organization of elements and the use of novel piezoelectric materials, have been the structural basis for improvements in 3DE matrix transducers.

#### Elements

Contemporary matrix-array transducers comprise as many elements in the elevational dimension as they do in the azimuthal dimension, with over 60 elements in each of these dimensions. While the elements are arranged in a two-dimensional grid, this array generates 3DE images. In order to be able to steer in the elevational plane, each element must be electrically independent from all other elements, and each element must be electrically active. The technology and electrical circuitry to electrically insulate and connect each element became commercially available in 2002. As a result, the contemporary matrix array transducer consists of thousands of electrically active elements that independently steer a scan line left and right, as well as up and down.

#### Piezoelectric Materials

The piezoelectric material in an ultrasound transducer is a fundamental determinant of system image quality. Piezoelectric transducer elements are responsible for delivery of ultrasound energy into the scanned tissue and for converting returning ultrasound echoes into electric signals. Their coupling efficiency in converting electrical energy to mechanical energy or vice versa is a key determinant of image quality, Doppler sensitivity and penetration. To create an overall piezoelectric effect, these elements must be subject to the application of an external electric field to align dipoles within polycrystalline materials. For almost forty years, a ceramic polycrystalline material, PZT (lead-zirconate-titanate) or PZT composites, has been the standard piezoelectric material used in medical imaging. This material is a uniform powder that is mixed with an organic binder; the resulting compound is baked into a dense polycrystalline structure. At its best, it achieves ~ 70% alignment of dipoles due to imperfect alignment of the individual dipoles. This leads to a corresponding constraint in the electromechanical coupling efficiency of the material.

**Figure 1:**
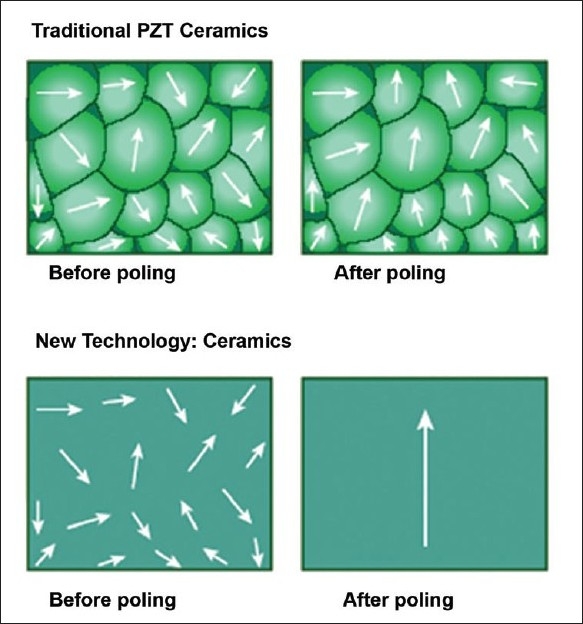
The top panel shows imperfect alignment of dipoles in traditional piezoelectric (PZT) crystals after poling (application of an external electrical field). The bottom panel shows almost perfect alignment of dipoles in new piezoelectric material

One example of new piezoelectric material involves growing crystals from a molten ceramic material, resulting in a homogenous crystal with fewer defects, lower losses and no grain boundaries.[8–10] When these crystals are poled at the preferred orientation(s), near perfect alignment of dipoles (~100%) is achievable resulting in dramatically enhanced electromechanical properties [Figure 1]. The efficiency of conversion of electrical to mechanical energy improves by as much as 68–85% when compared to PZT ceramics currently used in ultrasound transducers. The new piezoelectric materials provide increased bandwidth and sensitivity, resulting in both penetration and high-resolution. The improved arrangement of atoms in these new piezoelectric materials, and their superior strain energy density, translate into advances in transducer miniaturization. The recent implementation of these advances has led to the availability of a high frequency matrix 3DE transthoracic transducer that has dramatically enhanced the applicability of 3DE to pediatric populations.[11,12]

### Three-dimensional beam forming and steering

Beam forming constitutes the steering and focusing of transmitted and received scan lines. For 3DE, this means that beam former must be steered both in the azimuthal and elevational planes. This is achieved both in the ultrasound system and within the transducer itself, using highly specialized integrated circuits to create a 3D trapezoid of acoustic information that is processed. These 3DE data are summed, processed and finally placed into rectangular space using a 3D scan converter.

### Display of 3DE

**Figure 2:**
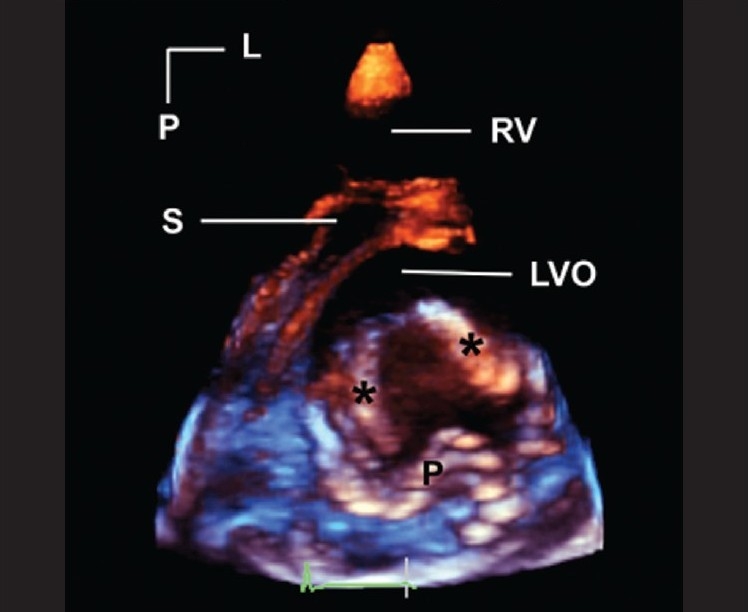
This is a parasternal short axis image of a cleft in the anterior mitral leaflet. Asterisks mark the edges of the cleft. A tissue colorization map has been applied to the image. This has the effect of coloring tissues in the near field (near to either the transducer or the front of the 3D image) orange. Tissues in the far filed are colored blue. This is a dynamic after-effect, which means that as the operator rotates, tilts or otherwise manipulates the image, the color effect correspondingly changes in real-time. L, left; LVO, left ventricular outflow tract; P, posterior; RV, right ventricle; S, septum

Two-dimensional computer displays consist of rows and blocks of picture elements, termed pixels, that comprise a 2D image. In contrast, a 3DE data set consists of bricks of pixels, termed volume elements or voxels. However, even for a 3DE data set, the two-dimensional nature of the display imposes restrictions on the ability to appreciate depth. As shown initially by the ancient Greeks and subsequently rediscovered during the Renaissance, perspective is used to simulate the appearance of 3D depth, providing objects the appearance of being close to or deeper / further away from the screen. The process of adding perspective is done by casting a light beam through the collection of voxels. The light beam either hits enough tissue so as to render it opaque, or it keeps shining through transparent voxels so as to render it transparent. More recent algorithms apply different hues to the front of the data set (nearest to the screen) as opposed to voxels that are far from the screen [Figure 2]. The user has the ability to rotate and tilt the data set on the computer screen. Tools are available to ‘cut away’ interfering structures, thus performing ‘virtual dissection’.

### Software for quantification

Quantification requires segmentation of structures of interest from the acquired data. Since myocardial motion occurs in three spatial dimensions, 2DE planes are inherently incapable of capturing the entire motion. Two-dimensional techniques for quantitation are based on geometric formulas that rely on assumptions regarding the shapes of cardiac structures. However, these assumptions are frequently incorrect. In contrast, 3DE acquisitions include the entire extent of the structure, thus minimizing the possibility of foreshortening of the apex or any geometric assumptions regarding shape. Three-dimensional quantitative software tools have the potential to quantify cardiac structures accurately regardless of their shape. Advances in the software tools for processing 3DE datasets have mirrored the rapid advances in transducer technology that have occurred over the past five years.

**Figure 3:**
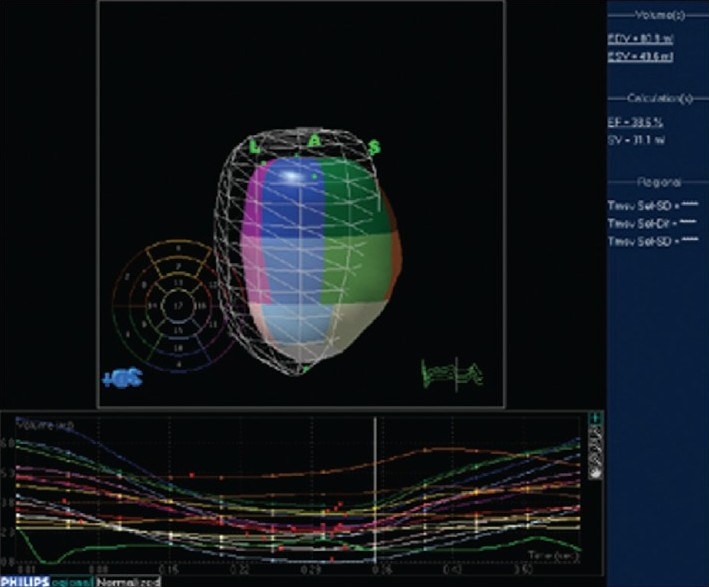
Software for processing 3DE enables quantitation of left ventricular volumes throughout the cardiac cycle, providing enddiastolic and end-systolic volume as well as ejection fraction. The mesh represents left ventricular volume at end-diastole. The cast of the left ventricular cavity consists of segments of varying colors, each of which represents a sub-volume of the ventricular cavity based on the American Society of Echocardiography 16-segment model. The change in volume of each sub-volume is represented graphically, with time on the X axis and volume on the Y axis

3DE volumetric techniques rely on definition of chamber cavities, that is, the blood-endocardium interface. The software constructs this interface by using a process known as surface rendering, and represents it as a mesh of points and lines. This software-generated mesh is calculated for every frame of acquisition, thus providing a moving cast of the cavity of the ventricle during the cardiac cycle. Since this is digital data, it provides for ease of computation of global and regional volumes, synchrony as well as parametric displays of endocardial excursion and timing of contraction [Figure 3].

**Figure 4:**
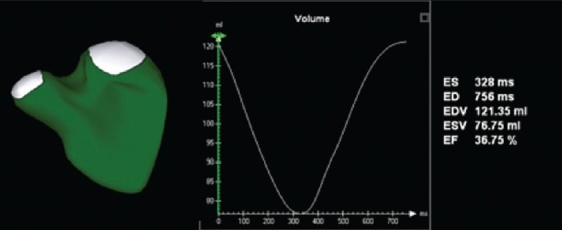
Novel software provides the ability to measure right ventricular volume throughout the cardiac cycle. This yields a surface-rendered model that is displayed as a mesh of lines and points. The change in volume is represented graphically, with time on the X axis and volume on the Y axis

3DE quantification tools for the left ventricle (LV) are more technologically advanced than for other cardiac structures. Until recently, 3DE LV quantification tools employed the method of disk summation. With improvements in computing speeds and programming, newer tools have been developed to provide instantaneous tracking of the blood pool - endocardium interface at each frame of acquisition. This provides a surface-rendered model that is displayed as a mesh of lines and points. However, these approaches are still based on some basic 3D geometric assumptions regarding left ventricular shape, and therefore their application cannot be extended to the right ventricle or to univentricular hearts. Given the complex shape and architecture of the right ventricle, it is not surprising that tools for quantifying right ventricular (RV) volume have been slower to mature. Until very recently, these tools utilized the method of disks for volumetrics.[13] Novel software now provides instantaneous tracking of the blood pool - endocardium interface at each frame of acquisition [Figure 4]. This yields a surface-rendered model that is displayed as a mesh of lines and points.[14]

Quantitative software for the mitral valve provides the ability to perform sophisticated analyses of the nonplanar shape of the mitral annulus and to measure 3D structures including annular diameters, commissural lengths, leaflet surface areas.[15,16] Quantitative techniques have also been developed to provide volumetric measurements of 3D color flow using non-aliased color flow data.

### Modes of 3DE

Electronically-steered 3DE systems have two major modes of scanning: live and EKG-gated. The live mode is the only one where the system scans in 3D real-time. A defining characteristic of this mode is: if the transducer comes off the chest, the image disappears. The live 3D mode can also be operated within a three-dimensionally shaped zoom box. Live 3DE modes provide narrow (20 to 30 degrees in the elevation plane) datasets that have high voxel density. Live 3DE can be obtained on patients with arrhythmias or with an active precordium with no potential for motion or stitch artifacts.

**Figure 5:**
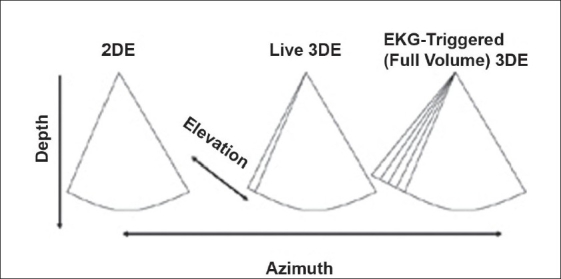
This figure depicts the differences between 2D, live 3D and full-volume (EKG-triggered) 3DE. Conventional 2DE is shown in the left panel. Live 3DE imaging (center panel) adds the elevation plane. The shape of the image is therefore trapezoidal rather than pie-shaped. The right panel depicts EKG-triggered (Full Volume) 3DE imaging, which provides a wider trapezoid

EKG-gated modes are required to provide wider volumes while maintaining adequate frame rates [Figure 5]. Gating allows for anywhere from four to eight smaller volumes to be stitched together to generate volumes that are greater than 90 degrees wide in the elevation plane, at frame rates exceeding 30 Hz. Gated modes have comparatively lower voxel density, and are subject to both motion and stitch artifacts. Recent enhancements have improved the ability to acquire gated full volume data among patients with arrhythmias. Gated modes are available using grayscale or with color flow Doppler.

Contemporary 3DE systems provide the user with tools to vary frame rate, 3D volume size and image resolution. Increasing the requirement in one of these causes a drop in the other two, all things being equal.

Given the potential for motion and stitch artifacts with gated modes and the need for high spatial and temporal resolution, it has been our practice to use live 3DE to delineate anatomy. We reserve the use of gated modes for the following:

- Complicated cardiac anatomy that requires extensive offline cropping
- Targets that do not fit within a live 3DE window
- Quantitation of chamber volumes
- 3DE color flow demonstrations of regurgitant jets or shunt flows.

### Modes

**Figure 6:**
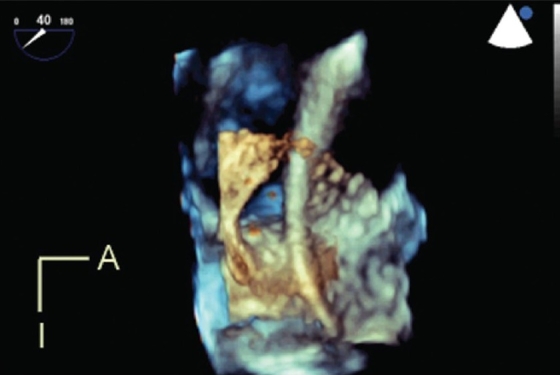
Live 3D transesophageal echocardiography demonstrates a catheter passing through a large atrial septal defect. The viewing perspective is unique: the observer is virtually located within the left atrium, looking rightwards. A, anterior; I, inferior

Live 3DE imaging has been commercially available for trans-thoracic and fetal applications since 2002. Live 3DE transesophageal echocardiographic imaging became commercially available in 2007. This has yielded images never before seen on the beating heart [Figure 6]. We anticipate that continuing improvements in transducer technology and the wider applicability of advances in piezoelectrics will enable the application of 3DE technology to an ever-increasing range of patient sizes, windows and applications.

### Clinical applications in congenital heart disease

3DE imaging has three broad areas of clinical application among patients with congenital heart disease: visualization of morphology, volumetric quantitation of chamber sizes and flows, and image-guided interventions.

### Visualization of morphology

Dating from an early stage in the development of 3DE technology, the structural complexity that is inherent to congenital heart disease has been identified as fertile substrate for exploration using 3DE.[17–19]

### The atrioventricular valves

3DE is valuable in delineating the morphology of the atrioventricular valves. Espinola-Zavaleta *et al*, described the role of 3DE in delineating congenital abnormalities of the mitral valve.[20] Lu *et al*, demonstrated that 3DE allowed comprehensive assessment of the anatomy of double orifice mitral valve.[21] Rawlins *et al*, demonstrated the additive value of 3DE and improved image quality using intraoperative epicardial 3DE to delineate the anatomy of atrioventricular (AV) valves.[22] Seliem *et al*, studied 41 patients with AV valve abnormalities and found that 3DE imaging was helpful in delineating the morphology of the valve leaflets and their chordal attachments, the subchordal apparatus, the mechanism and origin of regurgitation, and the geometry of the regurgitant volume.[23] Vettukatil *et al*, examined the role of 3DE in patients with Ebstein's anomaly of the tricuspid valve. They demonstrated that 3DE provided clear visualisation of the morphology of the valve leaflets, including the extent of their formation, the level of their attachment, and their degree of coaptation. They were also able to visualize the mechanism of regurgitation or stenosis.[24]

### Atrioventricular septal defect

**Figure 7:**
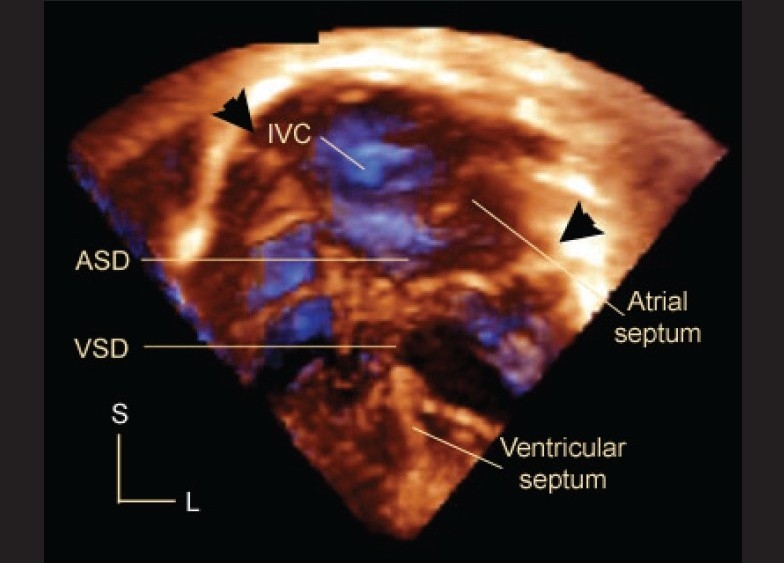
This is an apical four chamber 3D echocardiogram in a patient with atrioventricular septal defect. Arrowheads mark the hinges of the common atrioventricular junction. The bridging leaflets divide the defect into an interatrial (ASD) and interventricular (VSD) component. Note the posterior location of the inferior vena caval orifice (IVC) relative to the septal structures. L, left; S, superior

**Figure 8:**
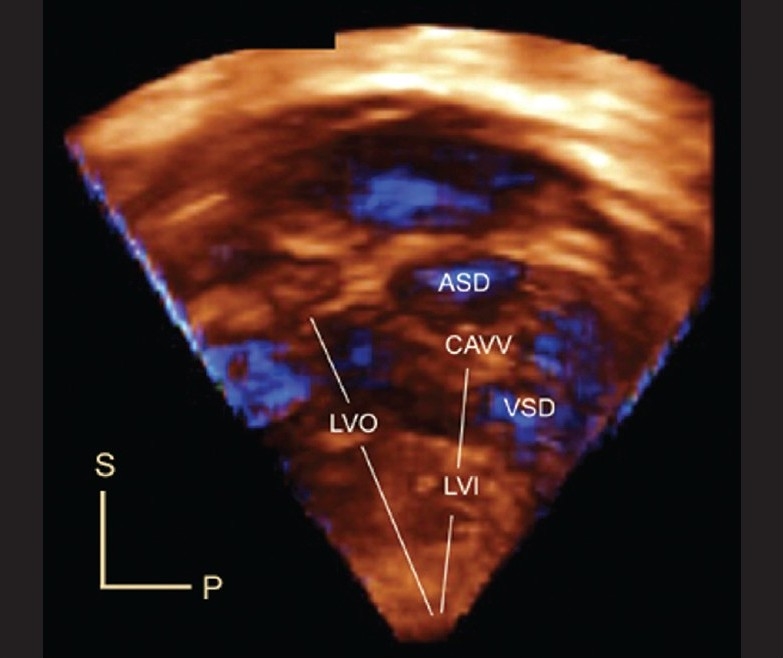
This is an en face view of the left ventricular aspect of the ventricular septum in a patient with atrioventricular septal defect. The free walls of the left atrium and left ventricle have been cropped. The viewer is looking from left to right. This view demonstrates the left ventricular inflow-outflow disproportion that characterizes AV septal defect. Note the crescentic inferior margin of the atrial septum, and the scooped-out edge of the interventricular septum. This view provides excellent anatomic detail regarding the relationships between the bridging leaflets of the common AV valve (CAVV) and the septal structures. P, posterior; S, superior

Hlavacek *et al*, studied 52 datasets on 51 patients with atrioventricular septal defects (AVSD) and showed that gated 3DE views could be cropped to obtain *en face* views of the atrial and ventricular septa.[25] These views provide a clear understanding of the relationships of the bridging leaflets to the septal structures [Figures 7,8]. These views have been useful to determine the precise location of the interventricular communication relative to the bridging leaflets, and to demonstrate how these relationships determine the level of shunting (atrial, ventricular or both atrial and ventricular). They found that 3DE on unrepaired balanced AVSD and repaired AVSD with residual lesions was more often additive/useful (33/36; 92%) than on repaired AVSD without residual lesions or unbalanced AVSD (9/16 (56%), P=0.009). 3DE was additive or useful in all 3 patients with unbalanced AVSD being considered for biventricular repair. Useful information obtained by 3DE included: precise characterization of mitral regurgitation and leaflet anatomy, substrate for subaortic stenosis, valve anatomy, and presence and location of additional septal defects.

### The atrial and ventricular septa

Tantengco *et al*, showed that 3DE reconstructions provided unique *en face* views of atrial and ventricular septal defects.[26] Cheng *et al*, studied 38 patients with atrial and / or ventricular septal defects using 3DE, and compared their results to 2DE and surgical findings. They demonstrated novel 3DE views of both atrial and ventricular septal defects and improved accuracy of quantification of the size of the defect by 3DE compared to 2DE (r = 0.92 vs. r = 0.69).[27] This approach has also been used to demonstrate the morphology of muscular ventricular septal defects.[28] We have found live 3DE to be of great value in evaluating complex malformations of the outflow tract that involve malalignment of the outlet septum.

### The aortic arch, pulmonary arteries and aortopulmonary shunts

**Figure 9:**
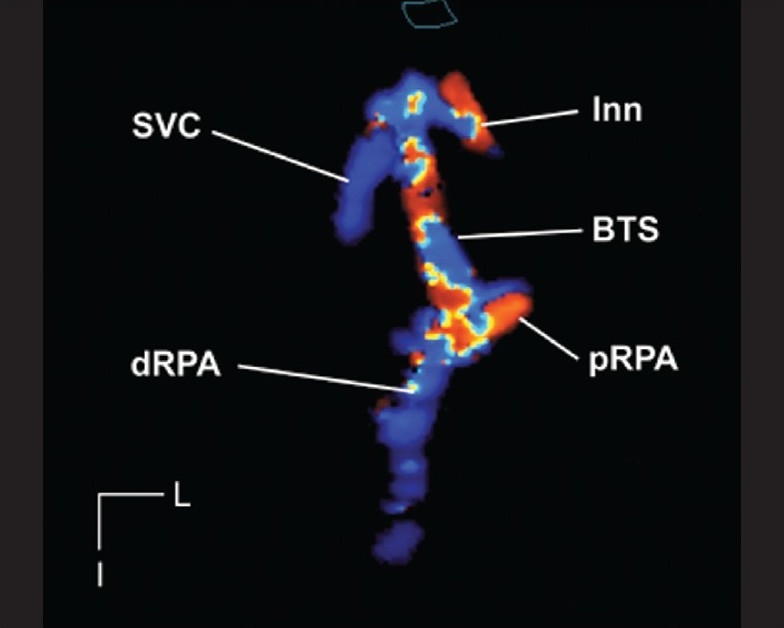
This is a 3DE color flow, ECG-triggered (full volume) acquisition demonstrating a right sided Blalock-Taussig shunt (BTS). The grayscale image has been suppressed, thus providing an echocardiographic angiogram. The shunt is seen in its entirety from its origin from the innominate artery (Inn) to its insertion. The proximal and distal right pulmonary artery (pRPA and dRPA respectively) are well seen. Note the proximity of the superior vena cava (SVC) to the cranial end of the shunt. This dataset can be rotated, tilted and examined in an infinite number of planes in order to delineate the location of stenosis. I, inferior; L, left

3DE color flow Doppler has been used to provide echocardiographic ‘angiograms’ of flow patterns in the aortic arch (coarctation of the aorta), the branch pulmonary arteries (the Lecompte maneuver) and across Blalock-Taussig shunts.[29] These authors examined echocardiographic ‘angiograms’ in 26 patients [Figure 9]. 3DE provided additional diagnostic information in 10 of 26 patients (38%). In 17 of 26 patients (65%), validation of the 3DE diagnosis was available at surgery, cardiac catheterization, MRI or CT angiography.

### The Aortic Valve and Outflow Tract

Sadagopan *et al*, examined the role of 3DE in 8 children who subsequently underwent surgery for congenital aortic valvar stenosis. They showed that 3DE was accurate in providing measurements of aortic valve annulus, number of valve leaflets, in identifying sites of fusion of the leaflets as well as nodules and excrescences that characterized dysplastic valves.[30] Bharucha *et al*, studied 16 patients with subaortic stenosis. Using a form of 3DE reconstruction known as multiplanar reconstruction, which provides access to an unlimited number of 2DE planes, they demonstrated abnormalities of mitral valve leaflet or chordal apparatus attachments (14 patients), abnormal ventricular muscle band (11 patients) and abnormal increased aorto-mitral separation (2 patients).[31]

### Characterization of Left Ventricular Noncompaction

Baker *et al*, evaluated 4 patients with left ventricular non-compaction using 3DE.[32] They found that 3DE enabled diagnosis and provided detailed characterization of the affected myocardium, including easy visualization of entire trabecular projections, intertrabecular recesses, endocardial borders and wall motion abnormalities of the affected myocardium. 3DE enabled easy differentiation between compacted and noncompacted portions of the myocardium.

### Quantitation of Chamber Dimensions, Valve Apparatus, Function and Flows

#### Left ventricular volumetrics

3DE quantitation provides both proven and potential value for pediatric echocardiography. Bu *et al*, compared 3DE measurements of LV volumetrics to those obtained using MRI.[33] Their study showed that 3DE measurements of LV end-systolic volume, end-diastolic volume, mass, stroke volume and ejection fraction in children using a rapid full volume acquisition strategy are feasible, reproducible and comparable with MRI measurements. They found good correlations between the two methods, but a tendency towards mild underestimation of volumes by 3DE. Interestingly, estimates of ejection fraction were in closer agreement. Baker *et al*, evaluated the feasibility of 3DE LV volumetrics, as well as the resource utilization, learning curve, inter- and intra-observer reproducibility of this technique. The study design involved 15 datasets and four observers who had varying degrees of (self-rated) experience with 3DE quantitation.[34] They found that in 59 of 60 instances, observers were able to obtain 3D LV ejection fraction in less than 3 minutes (median time 1 minute and 27 seconds). They demonstrated a learning curve for the observer with the lowest level of self-rated experience. Their study also showed excellent inter- and intra-observer reproducibility for 3DE LV volumetrics.

#### Left ventricular mass

Studies in adults have validated 3DE as an accurate method for measuring LV mass.[35–37] While studies in children have been limited in number and scope, Riehle *et al* recently demonstrated excellent correlations between LV mass measured by 3DE and MRI.[38]

#### Left ventricular dyssynchrony

The 3DE approach to measuring intra-LV dysynchrony utilizes the American Society of Echocardiography's 16-segment model of the LV.[39] It measures the time of each subvolume from maximal (end-diastolic) volume to minimal volume, and the standard deviation of these time intervals. The higher is the value of the standard deviation, the higher the implied degree of intra-LV dyssynchrony. Baker *et al*, examined the association between LV dysfunction and intra-LV dyssynchrony in children using 3DE.[40] They studied 9 children with dilated cardiomyopathy and an equal number of age- and body size-matched normals. They found that normal patients had 3DE dyssynchrony indices that were below 3%. Among children with dilated cardiomyopathy, there was a clear threshold value of LV ejection fraction (EF): at an LVEF below 35-40%, intra-LV dyssynchrony was the rule. In contrast, patients whose LVEF was higher than 40% exhibited no significant dyssynchrony.

#### Right ventricular volumetrics

Until recently, the accuracy of 3DE measurement of RV volume and ejection fraction had not been evaluated. This is unsurprising given the complex architecture of the right ventricle, and the technical difficulty of imaging it adequately. Recently, Gopal *et al*, reported a large series of adults who underwent RV volumetric measurements using MRI and 3DE using the disk summation method.[13] Niemann *et al*, studied RV volumes by 3DE using a new and robust protocol that utilizes multiplanar reconstruction and tracing with semi-automated border detection in 16 children with congenital heart disease.[14] They found excellent correlations between MRI and 3DE for measurement of RV volumes and ejection fraction. As these tools become more widely available, user-friendly and accurate, we may well see the emergence of a new paradigm in the use of echocardiographic parameters as surrogate outcome measures in clinical trials of medications and pacing strategies. The Pediatric Heart Disease Clinical Research Network is examining RV volumetrics as a secondary outcome measure in the Single Ventricle Reconstruction study, which examines for differences between initial shunt type on survival following the Norwood procedure for neonates with hypoplastic left heart syndrome (unpublished, http://www.pediatricheartnetwork.org/svrforhealthcareproviders.asp).

#### Visualization and quantitation of 3DE color flow

Multiplanar reconstruction tools provide the ability to not only visualize but also to trace and measure the area of valvar regurgitant orifices at the level of the vena contracta. While this technique is new and has not been validated, it has been shown to be feasible, and has already yielded new insights into the shape of regurgitant jets, and holds promise as a tool to enhance the echocardiographer's ability to serially quantify valve regurgitation.[41–43]

Pemberton *et al*, developed a technique for quantifying non-aliased 3DE color flow jets *in vitro*.[44] They validated this technique on open-chested pigs; when compared to measurements obtained from flow probes positioned on the ascending aorta, they obtained excellent correlations between the two methods.[45] They compared 143 individual measurements of cardiac output and found excellent correlation between the two techniques (r^2^ = 0.93). 3DE quantification of color flow has recently been validated in adults by comparison to cardiac outputs obtained by thermodilution.[46] Application of this technique to pathologic states could lead to potentially more accurate measurements of regurgitant volumes and fractions.[47]

### Image-guided interventions

**Figure 10:**
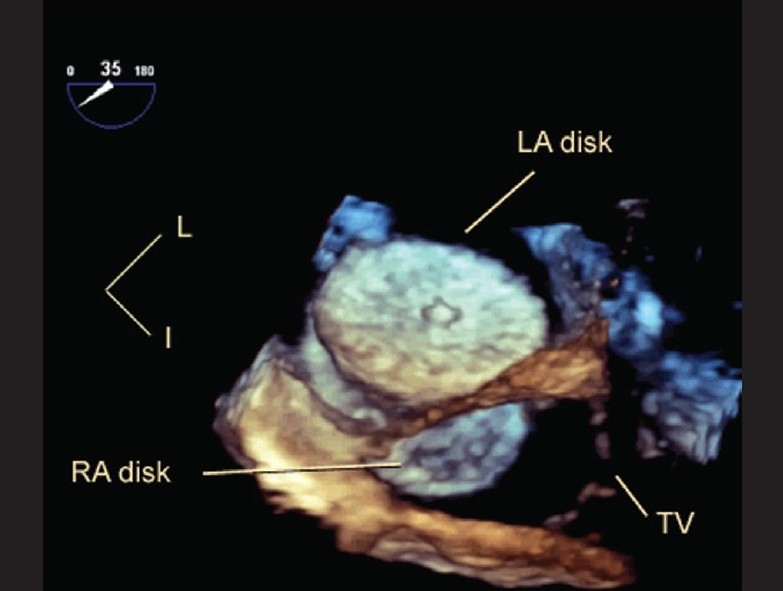
This is a live 3D transesophageal echocardiogram immediately following device closure of a fenestrated atrial septum with a cribriform Amplatzer septal occluder. The viewer is ‘virtually’ placed inside the left atrium, looking rightwards and posteriorly. The entire left atrial disk of the device is seen, with its central umbilication and the meshwork of metal that constitutes its frame. Part of the inferior portion of the right atrial disk (RA disk) and the tricuspid valve apparatus (TV) are also seen. I, inferior; L, left

**Figure 11:**
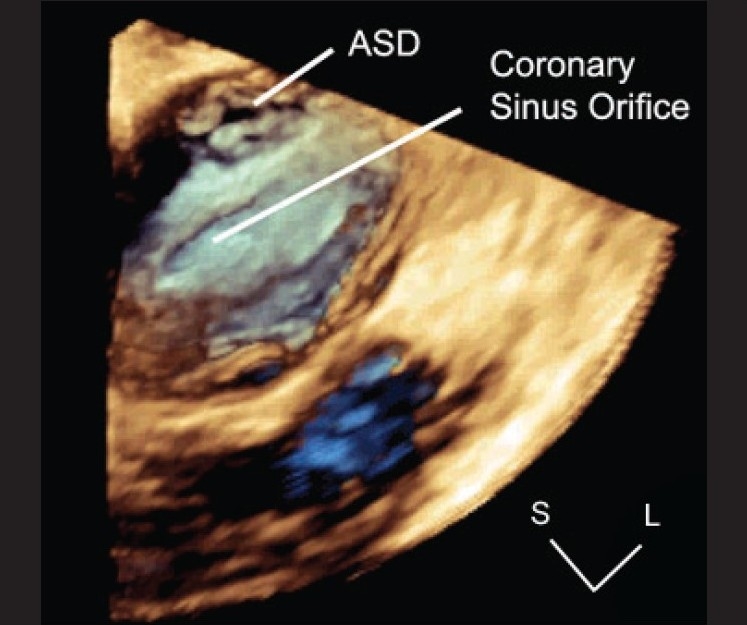
This is a live 3DE transesophageal echocardiogram in a patient with a low secundum atrial septal defect. 2DE imaging demonstrated that the defect was in proximity to the coronary sinus, raising doubts about candidacy for device closure. In this 3DE image, the free walls and appendage of the right atrium have been removed. The viewer is looking posteriorly and leftwards. Note the high resolution of anatomic detail. The atrial septal defect is separated from the coronary sinus orifice by a reasonable distance. Based on the shape and size of the defect, this was felt by 3DE to be a reasonable candidate for device closure, which was successfully accomplished. L, left; S, superior

The high temporal and spatial resolution of trans-thoracic and particularly trans-esophageal echocardiography has ignited interest in the potential uses of live 3DE to guide interventions. Scheurer *et al*, demonstrated the use of live 3DE to guide the performance of endomyocardial biopsy in children.[48] In their experience, the use of live 3DE guidance was associated with no complications, including no new tricuspid valve leaflet flail or pericardial effusion. 3DE proved to be a reliable noninvasive modality to accurately direct the bioptome to the desired site of biopsy within the right ventricle. As familiarity with this technique increased, the need for fluoroscopic guidance of bioptome manipulation in the right ventricle was minimized. Del Nido *et al*, have extended the concept of image-guided intervention to the very novel approach of epicardial live 3DE-guided, open-chest, closed-heart, off-bypass cardiac surgery.[49–52] They began with *in vitro* validation of the ability of live 3DE to guide the performance of common surgical tasks. More recently, they have undertaken closure of small atrial septal defects in the porcine model using live 3DE guidance. With the recent introduction of live 3D TEE, we have been able to obtain spectacular images of cardiac chambers, septal structures and valves. Live 3D TEE is being used to guide catheter manipulations, trans-septal procedures and closure of atrial septal defects[53] [Figures 10,11].

### Learning curve

The learning curve with 3DE is steep but negotiable. Our experience would suggest that the success of 3DE in a program requires advocacy and an investment of time by both echocardiographers and sonographers. The acceptance of 3DE is improving on a global level, albeit at an early stage of the technology cycle. We have developed and implemented an interactive teaching course that utilizes simulations using 3DE datasets with rehearsal and direct mentoring; this has been shown to be useful in overcoming the steep part of the learning curve.[54]

### Limitations

Many of the limitations of 2DE imaging extend to 3D echocardiography. Patients who have poor windows for 2DE examinations are unlikely to have good 3DE windows. Structures that are difficult to visualize by 2DE because they are thin and parallel to the imaging plane, such as the atrial septum in a teenager as viewed from an apical view, will probably remain difficult to visualize from that imaging window by 3DE.

Given the early stage of development of this technology at the current time, it is unsurprising that governing bodies such as the American Society of Echocardiography have not yet developed formal protocols.

### Future directions

Over the next decade, advances in the 3DE arena will involve technical enhancements such as improved image resolution, holographic displays, a wide range of validated software tools for quantification, and enhancements to work flow. New, multi-modality applications will increasingly bring 3DE into the mainstream. Refinements in transducer technology will make high-resolution 3DE available across the spectrum of patient sizes. We anticipate a 3DE TEE probe miniaturized for pediatric usage. With the growing interest in multi-modality imaging, 3DE volumetric data will eventually be integrated with the pressure data that is available during cardiac catheterization, yielding pressure-volume loops that can be obtained as a matter of routine clinical practice.

## References

[1] Tajik AJ, Seward JB, Hagler DJ, Mair DD, Lie JT (1978). Two-dimensional real-time ultrasonic imaging of the heart and great vessels: Technique, image orientation, structure identification and validation. Mayo Clin Proc.

[2] Ariet M, Geiser EA, Lupkiewicz SM, Conetta DA, Conti CR (1984). Evaluation of a three-dimensional reconstruction to compute left ventricular volume and mass. Am J Cardiol.

[3] Dekker DL, Piziali RL, Dong E (1974). A system for ultrasonically imaging the human heart in three dimensions. Comput Biomed Res.

[4] Linker DT, Moritz WE, Pearlman AS (1986). A new three-dimensional echocardiographic method of right ventricular volume measurement: *In vitro* validation. J Am Coll Cardiol.

[5] Matsumoto M, Matsuo H, Kitabatake A, Inoue M, Hamanaka Y, Tamura S (1977). Three-dimensional echocardiograms and two-dimensional echocardiographic images at desired planes by a computerized system. Ultrasound Med Biol.

[6] Von Ramm OT, Smith SW (1990). Real time volumetric ultrasound imaging system. J Digital Imaging.

[7] Salgo IS (2007). Three-dimensional echocardiographic technology. Cardiol Clin.

[8] Park SE, Shrout TR (1997). Characteristics of relaxor-based piezoelectric single crystals for ultrasonic transducers. IEEE Trans. Ultrason Ferroelectr Freq Control.

[9] Kuwata J, Uchino K, Nomura S (1982). Dielectric and piezoelectric properties of 0.91Pb(Zn1/3Nb2/O3-0.09PbTiO3 single crystals. Jpn J Appl Phys.

[10] Gururaja TR, Panda RK, Chen J (1999). Single crystal transducers for medical imaging applications. Ultrasonics Symposium Proc.

[11] Acar P, Abadir S, Paranon S, Latcu G, Grosjean J, Dulac Y (2007). Live 3D echocardiography with the pediatric matrix probe. Echocardiography.

[12] Simpson JM (2007). Real-time three-dimensional echocardiography of congenital heart disease using a high frequency paediatric matrix transducer. Eur J Echocardiogr.

[13] Gopal AS, Chukwu EO, Iwuchukwu CJ, Katz AS, Toole RS, Schapiro W (2007). Normal values of right ventricular size and function by real-time 3-dimensional echocardiography: Comparison with cardiac magnetic resonance imaging. J Am Soc Echocardiogr.

[14] Niemann PS, Pinho L, Balbach T, Galuschky C, Blankenhagen M, Silberbach M (2007). Anatomically oriented right ventricular volume measurements with dynamic three-dimensional echocardiography validated by 3-Tesla magnetic resonance imaging. J Am Coll Cardiol.

[15] Salgo IS, Gorman JH, Gorman RC, Jackson BM, Bowen FW, Plappert T (2002). Effect of annular shape on leaflet curvature in reducing mitral leaflet stress. Circulation.

[16] Watanabe N, Ogasawara Y, Yamaura Y, Wada N, Kawamoto T, Toyota E (2005). Mitral annulus flattens in ischemic mitral regurgitation: Geometric differences between inferior and anterior myocardial infarction: A real-time 3-dimensional echocardiographic study. Circulation.

[17] Vogel M, Losch S (1994). Dynamic three-dimensional echocardiography with a computed tomography imaging probe: Initial clinical experience with transthoracic application in infants and children with congenital heart defects. Br Heart J.

[18] Vogel M, Ho SY, Anderson RH (1995). Comparison of three dimensional echocardiographic findings with anatomical specimens of various congenitally malformed hearts. Br Heart J.

[19] Vogel M, Ho SY, Buhlmeyer K, Anderson RH (1995). Assessment of congenital heart defects by dynamic three-dimensional echocardiography: Methods of data acquisition and clinical potential. Acta Paediatr Suppl.

[20] Espinola-Zavaleta N, Vargas-Barron J, Keirns C, Rivera G, Romero-Cárdenas A, Roldán J (2002). Three-dimensional echocardiography in congenital malformations of the mitral valve. J Am Soc Echocardiogr.

[21] Lu Q, Lu X, Xie M, Wang X, Wang J, Yang Y (2006). Real-time three-dimensional echocardiography in assessment of congenital double orifice mitral valve. J Huazhong Univ Sci Technolog Med Sci.

[22] Rawlins DB, Austin C, Simpson JM (2006). Live three-dimensional paediatric intraoperative epicardial echocardiography as a guide to surgical repair of atrioventricular valves. Cardiol Young.

[23] Seliem MA, Fedec A, Szwast A, Farrell PE, Ewing S, Gruber PJ (2007). Atrioventricular valve morphology and dynamics in congenital heart disease as imaged with real-time 3-dimensional matrix-array echocardiography: Comparison with 2-dimensional imaging and surgical findings. J Am Soc Echocardiogr.

[24] Vettukattil JJ, Bharucha T, Anderson RH (2007). Defining Ebstein's malformation using three-dimensional echocardiography. Interact Cardiovasc Thorac Surg.

[25] Hlavacek AM, Chessa K, Crawford FA, Atz A, Shirali GS (2006). Real-time three-dimensional echocardiography is useful in the evaluation of patients with atrioventricular septal defects. Echocardiography.

[26] Tantengco MV, Bates JR, Ryan T, Caldwell R, Darragh R, Ensing GJ (1997). Dynamic three-dimensional echocardiographic reconstruction of congenital cardiac septation defects. Pediatr Cardiol.

[27] Cheng TO, Xie MX, Wang XF, Wang Y, Lu Q (2004). Real-time 3-dimensional echocardiography in assessing atrial and ventricular septal defects: An echocardiographic-surgical correlative study. Am Heart J.

[28] Mercer-Rosa L, Seliem MA, Fedec A, Rome J, Rychik J, Gaynor JW (2006). Illustration of the additional value of real-time 3-dimensional echocardiography to conventional transthoracic and transesophageal 2-dimensional echocardiography in imaging muscular ventricular septal defects: Does this have any impact on individual patient treatment?. J Am Soc Echocardiogr.

[29] Hlavacek A, Lucas J, Baker H, Chessa K, Shirali G (2006). Feasibility and utility of three-dimensional color flow echocardiography of the aortic arch: The “echocardiographic angiogram”. Echocardiography.

[30] Sadagopan SN, Veldtman GR, Sivaprakasam MC, Keeton BR, Gnanapragasam JP, Salmon AP (2006). Correlations with operative anatomy of real time three-dimensional echocardiographic imaging of congenital aortic valvar stenosis. Cardiol Young.

[31] Bharucha T, Ho SY, Vettukattil JJ (2008). Multiplanar review analysis of three-dimensional echocardiographic datasets gives new insights into the morphology of subaortic stenosis. Eur J Echocardiogr.

[32] Baker GH, Pereira NL, Hlavacek AM, Chessa K, Shirali G (2006). Transthoracic real-time three-dimensional echocardiography in the diagnosis and description of noncompaction of ventricular myocardium. Echocardiography.

[33] Bu L, Munns S, Zhang H, Disterhoft M, Dixon M, Stolpen A (2005). Rapid full volume data acquisition by real-time 3-dimensional echocardiography for assessment of left ventricular indexes in children: A validation study compared with magnetic resonance imaging. J Am Soc Echocardiogr.

[34] Baker GH, Flack EC, Hlavacek AM, Chessa KS, Fleming DM, Scheurer MA (2006). Variability and resource utilization of bedside three-dimensional echocardiographic quantitative measurements of left ventricular volume in congenital heart disease. Congenital Heart Dis.

[35] Mor-Avi V, Sugeng L, Weinert L, MacEneaney P, Caiani EG, Koch R (2004). Fast measurement of left ventricular mass with real-time three-dimensional echocardiography: Comparison with magnetic resonance imaging. Circulation.

[36] Pouleur AC, le Polain de Waroux JB, Pasquet A, Gerber BL, Gerard O, Allain P (2007). Assessment of left ventricular mass and volumes by three-dimensional echocardiography in patients with or without wall motion abnormalities: Comparison against cine magnetic resonance imaging. Heart.

[37] Caiani EG, Corsi C, Zamorano J, Sugeng L, MacEneaney P, Weinert L (2005). Improved semiautomated quantification of left ventricular volumes and ejection fraction using 3-dimensional echocardiography with a full matrix-array transducer: Comparison with magnetic resonance imaging. J Am Soc Echocardiogr.

[38] Riehle TJ, Mahle WT, Parks WJ, Sallee D, Fyfe DA (2008). Real-time three-dimensional echocardiographic acquisition and quantification of left ventricular indices in children and young adults with congenital heart disease: Comparison with magnetic resonance imaging. J Am Soc Echocardiogr.

[39] Kapetanakis S, Kearney MT, Siva A, Gall N, Cooklin M, Monaghan MJ (2005). Real-time three-dimensional echocardiography: A novel technique to quantify global left ventricular mechanical dyssynchrony. Circulation.

[40] Baker GH, Hlavacek AM, Chessa KS, Fleming DM, Shirali GS (2008). Left ventricular dysfunction is associated with intraventricular dyssynchrony by 3-dimensional echocardiography in children. J Am Soc Echocardiogr.

[41] Sugeng L, Weinert L, Lang RM (2007). Real-time 3-dimensional color Doppler flow of mitral and tricuspid regurgitation: Feasibility and initial quantitative comparison with 2-dimensional methods. J Am Soc Echocardiogr.

[42] Matsumura Y, Fukuda S, Tran H, Greenberg NL, Agler DA, Wada N (2008). Geometry of the proximal isovelocity surface area in mitral regurgitation by 3-dimensional color Doppler echocardiography: Difference between functional mitral regurgitation and prolapse regurgitation. Am Heart J.

[43] Sugeng L, Spencer KT, Mor-Avi V, DeCara JM, Bednarz JE, Weinert L (2003). Dynamic three-dimensional color flow Doppler: An improved technique for the assessment of mitral regurgitation. Echocardiography.

[44] Pemberton J, Hui L, Young M, Li X, Kenny A, Sahn DJ (2005). Accuracy of 3-dimensional color Doppler-derived flow volumes with increasing image depth. J Ultrasound Med.

[45] Pemberton J, Li X, Karamlou T, Sandquist CA, Thiele K, Shen I (2005). The use of live three-dimensional Doppler echocardiography in the measurement of cardiac output: An in vivo animal study. J Am Coll Cardiol.

[46] Lodato JA, Weinert L, Baumann R, Coon P, Anderson A, Kim A (2007). Use of 3-dimensional color Doppler echocardiography to measure stroke volume in human beings: Comparison with thermodilution. J Am Soc Echocardiogr.

[47] Pemberton J, Ge S, Thiele K, Jerosch-Herold M, Sahn DJ (2006). Real-time three-dimensional color Doppler echocardiography overcomes the inaccuracies of spectral Doppler for stroke volume calculation. J Am Soc Echocardiogr.

[48] Scheurer M, Bandisode V, Ruff P, Atz A, Shirali G (2006). Early experience with real-time three-dimensional echocardiographic guidance of right ventricular biopsy in children. Echocardiography.

[49] Suematsu Y, Martinez JF, Wolf BK, Marx GR, Stoll JA, DuPont PE (2005). Three-dimensional echo-guided beating heart surgery without cardiopulmonary bypass: Atrial septal defect closure in a swine model. J Thorac Cardiovasc Surg.

[50] Suematsu Y, Marx GR, Stoll JA, DuPont PE, Cleveland RO, Howe RD (2004). Three-dimensional echocardiography-guided beating-heart surgery without cardiopulmonary bypass: A feasibility study. J Thorac Cardiovasc Surg.

[51] Suematsu Y, Marx GR, Triedman JK, Mihaljevic T, Mora BN, Takamoto S (2004). Three-dimensional echocardiography-guided atrial septectomy: An experimental study. J Thorac Cardiovasc Surg.

[52] Vasilyev NV, Martinez JF, Freudenthal FP, Suematsu Y, Marx GR, del Nido PJ (2006). Three-dimensional echo and videocardioscopy-guided atrial septal defect closure. Ann Thorac Surg.

[53] Baker GH, Shirali GS, Bandisode V (2007). Transseptal left heart catheterization for a patient with a prosthetic mitral valve using live three-dimensional transesophageal echocardiography. Pediatr Cardiol.

[54] Jenkins C, Monaghan M, Shirali G, Guraraja R, Marwick TH (2007). An intensive interactive course for 3D echocardiography: Is "Crop Till You Drop" an effective learning strategy?. Eur J Echocardiogr.

